# Paper-Based Device for the Colorimetric Determination of Glucose in Whole-Blood Samples Using a Smartphone

**DOI:** 10.3390/bioengineering12111250

**Published:** 2025-11-15

**Authors:** Lara B. A. Boga, Katia Gianni, Mariano N. Aleman, Marcos S. Almirón Arroyo, Rossana E. Madrid

**Affiliations:** 1 Laboratorio de Medios e Interfases (LAMEIN), DBI, FACET, Universidad Nacional de Tucumán, Instituto Superior de Investigaciones Biológicas (INSIBIO), CONICET, Av. Independencia 1800, San Miguel de Tucumán 4000, Argentina; labian2013@gmail.com (L.B.A.B.); ka20_04@hotmail.com (K.G.); chato9595@gmail.com (M.S.A.A.); 2 Facultad de Bioquímica, Química y Farmacia, Universidad Nacional de Tucumán, Ayacucho 449, San Miguel de Tucumán 4000, Argentina; mariano_edu@hotmail.com

**Keywords:** POC devices, paper-based biosensors

## Abstract

In many clinical settings, there is a great need for rapid, simple, and reliable diagnostic tools for the detection and quantification of various biomarkers. These tools enable early medical decisions, which can significantly influence patient recovery. Paper-based analytical devices (PADs) have become promising platforms for rapid and low-cost diagnostic testing in recent years. Among the most important biomarkers is glucose, a key metabolite involved in numerous physiological processes, which allows for the diagnosis and control of diabetes, the prevention of serious long-term complications such as cardiovascular disease, and the monitoring of the effect of medication, diet, and exercise on sugar levels in these patients. A fundamental step in detecting this marker in laboratories is the separation of plasma from whole blood. Several studies have demonstrated the successful integration of plasma separation in μPADs. This work presents the development of a paper-based device for the colorimetric detection of glucose in whole-blood samples, allowing plasma separation and using a smartphone to perform quantitative determination.

## 1. Introduction

Whole-blood analysis is widely recognized as the gold standard in clinical diagnostics, offering a comprehensive assessment of a patient’s health status. However, the intense red coloration of hemoglobin can interfere with diagnostic techniques that rely on optical or colorimetric detection. Consequently, plasma separation from whole blood is often necessary—a process traditionally achieved through centrifugation. While effective, centrifugation requires specialized and bulky equipment, which limits its use in low-resource settings where laboratory infrastructure is not available or for Point of Care (POC) testing.

In many clinical scenarios, there is a pressing need for diagnostic tools that are rapid, simple, and reliable for the detection and quantification of biomarkers. These tools enable early medical decision-making, which can significantly influence patient outcomes. Delays in biomarker analysis may lead to increased risks of complications, long-term disability, or even death. Within this context, biosensors designed for Point-of-Care (POC) testing have gained considerable attention due to their ability to support timely and informed clinical decisions [1].

Among the most critical biomarkers is glucose, a key metabolite involved in numerous physiological processes. Blood glucose levels are essential indicators in diseases such as diabetes, where continuous monitoring is crucial for effective disease management. Additionally, low glucose levels can result in hypoglycemia, a condition triggered by extended fasting or intense physical activity. Therefore, accurate and frequent glucose monitoring is vital for both diagnosis and the prevention of complications related to hyperglycemia and hypoglycemia [2].

To support the development of accessible and effective diagnostic tools, the World Health Organization (WHO) established the ASSURED criteria for POC technologies: Affordable, Sensitive, Specific, User-friendly, Rapid and Robust, Equipment-free, and Deliverable to end users. These guidelines are particularly important in developing regions, where cost and ease of use are key factors driving the adoption of diagnostic innovations [3,4].

In this context, paper-based analytical devices (PADs) have emerged as promising platforms for low-cost, rapid diagnostic testing. Thanks to their porous structure and the ability to transport fluids through capillary action, paper is an ideal material for creating devices that operate without external pumps or instrumentation. Since their introduction by the Whitesides group in 2007—using photolithographic patterning techniques—microfluidic paper-based analytical devices (µPADs) have demonstrated significant potential in fields ranging from clinical diagnostics and environmental monitoring to food safety and biohazard detection [5,6].

Among the detection methods integrated into PADs, colorimetric assays are particularly appealing due to their ability to visually display results without requiring complex equipment. Although commercial test strips offer qualitative or semi-quantitative analysis, they often lack the precision and reproducibility needed for clinical reliability. While colorimeters can enhance measurement accuracy, they are typically expensive and require trained personnel to operate [7,8].

Beyond colorimetric sensing, smartphone integration has enabled alternative diagnostic modalities that exploit embedded cameras, inertial sensors, and artificial intelligence algorithms to extract physiological information without dedicated laboratory instruments. Examples include contactless optical detection of blood oxygen saturation using facial imaging [9], biomechanical analysis of movement through smartphone inertial sensors coupled with machine learning [10], and AI-driven mobile applications for pediatric diagnostics [11]. These studies collectively demonstrate the versatility of smartphones as biomedical platforms, extending well beyond traditional colorimetric biosensing.

Recent advances in technology have enabled the use of smartphones as powerful diagnostic tools at the point-of-care [12,13]. With high-resolution cameras and robust processing capabilities, smartphones have facilitated the development of mobile biosensors that overcome many limitations of traditional colorimetric devices—providing more affordable, portable, and user-friendly solutions for detecting biomarkers in blood and other biological fluids [14,15].

A critical step in many diagnostic assays is the separation of plasma from whole blood, as plasma contains essential biomarkers that reflect physiological and pathological states. Studies have demonstrated the successful integration of plasma separation into μPADs through different mechanisms [16,17]. One approach involves using blood separation membranes, such as LF1, which effectively trap blood cells while allowing plasma to wick into detection zones, enabling protein quantification in under two minutes [18]. Another strategy leverages red blood cell (RBC) agglutination, where antibodies immobilized on the paper matrix retain RBCs at the sample site, allowing clean plasma to migrate toward colorimetric readout zones [19,20]. Both methods have demonstrated the ability to deliver plasma with high purity and sufficient yield for accurate detection, achieving results comparable to conventional laboratory techniques. These integrated designs, combined with smartphone-based detection, represent a significant advancement in the development of fully self-contained, low-cost diagnostic tools suitable for resource-limited settings.

Another major challenge in the development of μPADs for electrochemical biomarker detection is the ability to collect sufficient plasma volume while preserving the biomarker integrity. A solution to this was proposed in a μPAD designed for the quantification of the S100B protein biomarker, relevant in neurological diagnostics [21]. Using NaCl-functionalized VF2 paper for blood collection and MF1 paper for plasma retention, the device achieved efficient plasma separation (50 µL from 300 µL of whole blood) in under four minutes [22].

This work presents the development of a paper-based device for colorimetric detection of glucose in whole-blood samples, using a smartphone for quantitative determination.

## 2. Materials and Methods

### 2.1. Substrate Selection and Characterization

In order to select the most suitable material for the separation of plasma from whole blood, a comparative evaluation was carried out between different types of paper available in the laboratory and those reported in the literature [22,23].

The materials evaluated were as follows:

- Whatman Paper N°1 (Cytiva, Little Chalfont, UK): with a pore size of 11 µm and a thickness of 180 µm. Its intermediate porous structure allows partial retention of blood cells, especially leukocytes, and provides a good balance between flow and mechanical support.
- Polycarbonate membrane (Isopore™; MilliporeSigma, Cork, Ireland): with 3 µm pores and 22 µm thickness. Its small pore size makes it suitable for the retention of erythrocytes and platelets, allowing cleaner separation of plasma.
- Glass fiber membrane (Cobetter Filtration Equipment Co., Ltd., Hangzhou, China): with a pore size of 3 µm and a thickness of 785 µm. Despite sharing the same pore size as the previous one, its greater thickness provides higher absorption and retention capacity, making it suitable for complementing the separation process.

### 2.2. Paper Functionalization and Plasma Separation

To facilitate selective plasma separation, the selected paper strips were functionalized with sodium chloride (NaCl) and sodium heparin (NORTHIA, C.A.B.A., Argentina) (25,000 IU/5 mL).

The heparin solution was diluted 1:100 in distilled water to obtain a final concentration of 250 IU/mL, representing approximately 0.074 M sodium [24]. In parallel, a stock solution of 1 M NaCl was prepared, and three different concentrations were obtained by serial dilution: 1 M, 0.68 M, and 0.154 M.

### 2.3. Colorimetric Enzymatic Assay for Glucose Detection

A colorimetric assay based on enzymatic reactions was used for glucose detection. The system included the enzymes glucose oxidase (GOx) from Aspergillus Níger type VII (100 units/g) and horseradish peroxidase (HRP) type VI of Armoracia rusticana (≥250 U/mg), both purchased from Thermo Fisher Scientific (Waltham, MA, USA), and the chromogenic reagent *TMB* (3,3′,5,5′-tetramethylbenzidine) [25].

The reactions involved were as follows:

(1) $$Glucose+\;O_{2}\;\to \;Gluconic\;Acid\;+\;H_{2}O_{2}\;$$

(2) $$H_{2}O_{2}+TMB(colorless)\;\to \;[TMB{]}^{ox}(blue)+H_{2}O$$

A 1.5 μL solution of chitosan (1 mg/mL) in 0.25% (*v*/*v*) acetic acid was applied, and the paper was air-dried at room temperature for approximately 10 min. Subsequently, a bi-enzymatic colorimetric reaction system, composed of glucose oxidase (GOx), horseradish peroxidase (HRP), and chromogenic reagents, was employed for detection. For enzyme immobilization, a simple drop-casting method was used. All necessary reagents were immobilized individually onto the chitosan-modified (Sigma-Aldrich, St. Louis, MO, USA) paper in a layer-by-layer manner, with a drying period of 10 min between each addition, in the following order:

- A 1.2 μL layer of *TMB* solution (pre-prepared).
- A 1.5 μL layer of HRP (7.11 U/mL) was prepared in phosphate-buffered saline (PBS, pH 7.4).
- A 1.5 μL layer of GOx (2 mg/mL) prepared in PBS (pH 7.4).
- A final 1.5 μL layer of *TMB* to complete the multilayer detection platform.

For glucose colorimetric analysis, specific enzymatic reactions were carried out between the analyte and the corresponding oxidases, generating hydrogen peroxide (*H*_2_*O*_2_). The resulting *H*_2_*O*_2_ further oxidized the co-immobilized chromogenic reagent (*TMB*), yielding colored products such as oxidized *TMB*, with HRP acting as the catalyst [26]. This allowed direct visual inspection and semi-quantitative analysis by the naked eye. For more sensitive quantitative analysis, color images of the test papers were captured using a smartphone via a specially designed app. Whole-blood tests were conducted to determine the optimal device design with blood sample volumes of 20, 15, and 8 μL.

### 2.4. Glucose Calibration

To obtain a reference value, an enzymatic glucose kit (Winer Lab^®^, Rosario, Argentina) was used, providing a known glucose concentration, which was related to the color intensity obtained in the colorimetric analysis. This analysis allowed the creation of an equation that automated glucose measurement in the samples.

### 2.5. Measurements Validation

Whole-blood samples were collected from participants by venipuncture between 8 and 10 AM, after an 8 h fast. The blood was drawn into heparinized tubes (TUBLOOD® S.A., C.A.B.A., Autónoma de Buenos Aires, Argentina). The plasma was then separated by centrifugation and assayed for fasting blood glucose using a Mindray BS-380 chemistry analyzer (Mindray Bio-Medical Electronics CO., Ltd., Shenzhen, China). All specimens were analyzed within a maximum of 2 h from collection and were qualitatively assessed for hemolysis. Parallel measurements of whole-blood samples and plasma from the same samples (centrifuged blood) were performed to validate that the measurements obtained with the chips are equivalent to glucose measurements normally performed in biochemical laboratories. The experiments were approved by the Ethical Committee of the Faculty of Medicine, Tucumán University, Tucumán, Argentina (Exp. No. 80509/2025).

### 2.6. Software Design for Glucose Detection

The app was developed using the library OpenCV. To use the app, the user must access the URL through the browser: https://detector-glucosa.netlify.app/ (accessed on 13 December 2024), where they must register to create a personal account and securely manage their glucose data. Once registered, the user logs in and can access their glucose records, as well as view a graph showing the evolution of their levels over time. In case of consistently high glucose levels, the user should consult a specialist so that the application can determine the appropriate and adjusted ranges for each user.

After logging in, the user can measure their glucose by uploading a photo of the test chip. The app allows capturing the image with the device’s camera or selecting an image from the gallery. The captured photo will be automatically analyzed, and the glucose level will be calculated, updating the corresponding graph.

**Figure 1:**
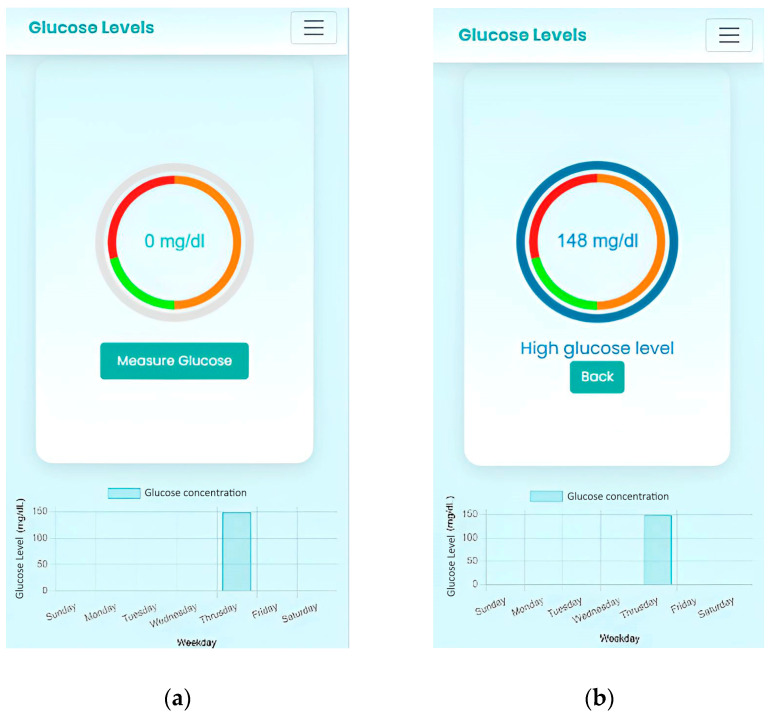
(**a**) Home page; (**b**) sample analysis and chart update. The gray circle indicates that no reading has been performed yet. After the image is analyzed, the outer circle is made complete and it’s colored with different shades of blue based on the glucose concentration level. If the concentration is low (orange zone), it’ll be light blue and will only be made complete up to a certain level in that zone. If the concentration is normal, it will be filled to some point in the green zone and will take on a darker shade of blue, and if the concentration is high, the circle will be filled to the red range and will take on an even darker shade of blue.

The app classifies glucose levels into “Low Glucose” (yellow), “Normal” (green), and “High Glucose” (red), and displays the graph with the corresponding concentration, as can be seen in Figure 1b. The system also allows viewing representative colors of glucose concentrations and downloading a PDF file with this information for use outside the application. The device allows for a visual comparison between the detected color and a reference scale that represents different glucose concentration ranges. This way, the user can interpret the results even without a Wi-Fi connection or access to a mobile phone.

### 2.7. Image Processing and Colorimetric Analysis

The software includes a module to detect the color resulting from the reaction between the glucose present in serum and the chromogenic reagent. For this purpose, the HSV (Hue, Saturation, Value) color model was used, which allows the isolation of specific shades associated with the presence of glucose [27,28].

#### 2.7.1. Step 1: Definition of HSV Ranges

The following color ranges were established for the detection of blue and green, expressed as HSV matrices: Blue: low range = ([90, 50, 50]), upper range = ([130, 255, 255]); Green: low range = ([35, 50, 50]), upper range = ([85, 255, 255]). These ranges allow the identification of both intense and soft shades of the target colors. In each set of brackets, the three numerical values represent the components of the HSV color model: Hue (H), Saturation (S), and Value (V), respectively. The Hue corresponds to the dominant wavelength of the color and is expressed in degrees on the color wheel (ranging from 0° to 360°), defining the perceived color tone (e.g., red, green, blue). The Saturation indicates the purity or intensity of the color, describing the degree to which it differs from a neutral gray, with higher values representing more vivid colors. The Value, also known as brightness, it represents the relative lightness or darkness of the color, with higher values corresponding to lighter shades.

#### 2.7.2. Step 2: Object Segmentation and Mask Generation

The image uploaded by the user is processed to identify the region of interest. Once the object is segmented, a binary (grayscale) mask is generated based on the defined HSV ranges.

#### 2.7.3. Step 3: Color Intensity Calculation

The intensity of the colorimetric reaction was quantified by counting the white pixels in the generated mask. This number is proportional to the presence of the desired color and, therefore, to the glucose concentration in the sample.

#### 2.7.4. Step 4: Quantitative Analysis

A linear regression was constructed to correlate the intensity values obtained with known glucose concentrations, using an enzymatic glucose kit as a reference. This relationship allows the interpretation of the results of the visual analysis in terms of actual concentration, facilitating the quantification of plasma glucose.

## 3. Results

For the comparative evaluation of the different types of paper, the main criterion was the ratio of paper pore size to the cellular dimensions of the blood components, primarily erythrocytes (~7 µm), leukocytes (8–20 µm), and platelets (~3 µm). Both pore size and paper thickness were considered critical parameters to ensure efficient cell retention without compromising capillary flow.

Different membrane materials were evaluated to optimize plasma separation: polycarbonate (3 µm pore size), glass fiber (3 µm pore size, 785 µm thickness), and Whatman No. 1 filter paper (11 µm pore size, 180 µm thickness). The polycarbonate membrane retained excessive sample due to platelet accumulation, which blocked capillary flow. The glass fiber membrane showed higher absorption capacity but required larger sample volumes and exhibited slower flow because of its high liquid retention.

When using whole blood, it contains many formed elements, and this probably clogs the pores, which is why it was very difficult for whole blood to flow through those papers. It is important to note that when using whole blood, separation must be rapid to prevent clotting. Therefore, if the flow is stopped by the accumulation of cells, the sample clots, and the device is unable to separate the blood.

When using heparinized blood, the effect is the opposite. Everything passes through the pores, as can be seen in Figure S1 in the Supplementary Material. For this reason, Whatman No. 1 paper was used despite having a pore size of 11 µm. The use of NaCl allows the erythrocytes, when crenated, to change their globular shape to a structure with spicules. These tend to accumulate and become trapped in the structure of the paper. Nilghaz et al. used, for example, Whatman No. 4 paper, with a pore size of 20–25 µm, and also managed to separate and retain the erythrocytes in the structure of the paper [23]. Whatman No. 1 provided the best balance between capillary velocity and partial cell retention, resulting in a stable flow. This paper proved to be the most efficient compared to the other materials evaluated.

The combined action of both agents, NaCl and sodium heparin, seeks to induce cellular deformation and prevent coagulation [29]. NaCl generates an osmotic gradient that causes erythrocyte crenation, favoring their aggregation and arrest in the initial areas of the paper; meanwhile, sodium heparin acts as an anticoagulant by inhibiting the action of thrombin [23].

The drop-casting method was selected for enzyme immobilization. Although there are advanced methods, such as covalent immobilization of enzymes, this simple procedure was chosen to avoid possible loss of enzyme activity that such complex processes can cause. The use of chitosan as a biopolymer was also explored to improve the distribution of enzymes on paper. Its ability to form uniform films and its compatibility with proteins position it as a promising candidate for further optimization [30].

One of the main challenges associated with paper-based colorimetric assays is the poor color uniformity across detection zones, which is mainly attributed to the uncontrolled flow of reagents [29]. Several strategies have been reported to enhance color uniformity in these colorimetric assays, such as the covalent immobilization of enzymes. Despite the advantages offered by these strategies, their application has not been universal due to potential enzyme inactivation and the complexity of multiple experimental steps. Chitosan is a biocompatible and biodegradable substance with excellent film-forming ability and a high specific surface area. Paper modification with chitosan has been shown to improve color uniformity in such assays [23].

*TMB* is a chromogenic substrate commonly used in biochemical assays such as ELISA. Upon oxidation, it produces a blue color, making it an ideal choice for differentiation from blood. However, *TMB* is sensitive to environmental factors such as pH and the presence of certain metal ions, which can act as catalysts or inhibitors in redox reactions.

To improve performance, the cellulose-based detection zones were modified with chitosan, leveraging electrostatic interactions between the negatively charged cellulose and the positively charged chitosan, as well as additional interactions such as hydrogen bonding.

**Figure 2:**
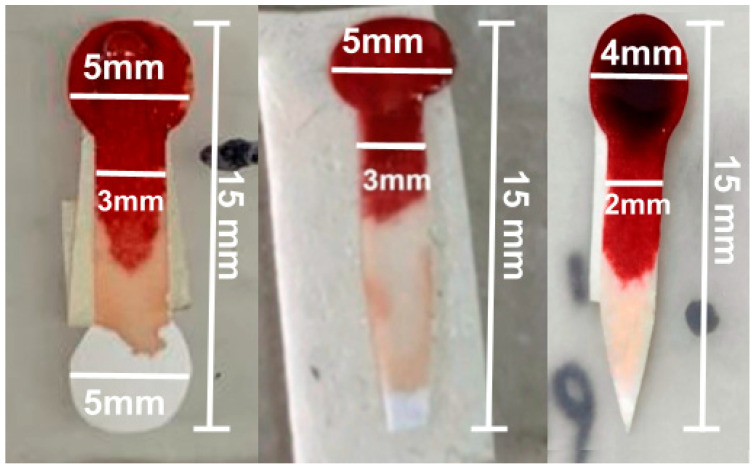
The result of the serum separation process with the different configurations of test strips.

The osmotic action induced by NaCl favors the physical retention of red blood cells in the paper fibers, facilitated by the compression of the electrical double layer surrounding the cells, which increases interactions with the cellulose matrix. Concentrations were selected so as not to compromise the integrity of cellular components. This passive serum separation occurs by capillarity in less than five minutes and without the need for external centrifugation devices. The following figure shows the result of the separation:

The final design included a 5 mm diameter sample collection zone and an analysis zone, connected by a 10 mm long and 3 mm wide channel, but the plasma distribution in the analysis zone was uneven. To improve this distribution, the diameter of the test zone was reduced to 4 mm, and the channel was narrowed to 2 mm, allowing for greater uniformity in plasma distribution for colorimetric analysis, as shown in Figure 2.

The improved design introduced a pointed termination at the end of the analysis zone, which concentrates the plasma flow and promotes a more uniform distribution across the detection area. This geometry ensures that the filtered plasma reaches the analysis zone in a controlled manner, minimizing accumulation or gaps that were observed in the previous circular design, as illustrated in Figure 2. Another important point to take into account is the sample volume. The goal was to minimize it to prioritize its use as a POC device, so that the sample could be obtained from a single drop of blood, without the need for extraction. The pointed shape thus serves a functional role in optimizing capillary flow of minimal sample volume within the device, rather than being a mere geometric modification.

During whole-blood tests, the effectiveness of different saline concentrations in separating blood plasma was evaluated (0.15 M, 0.68 M, and 1 M). The 1 M concentration was the most effective, achieving adequate plasma separation without hemoglobin contamination. Additionally, sodium heparin was incorporated into the paper to improve plasma flow and prevent clotting. The tests also evaluated the use of smaller blood volumes, adapting the design to work with smaller samples, which is crucial for Point-of-Care applications. The best concentration was 1 M, which was used for all subsequent measurements. These results can be seen in a congress communication of our group [31].

**Figure 3:**
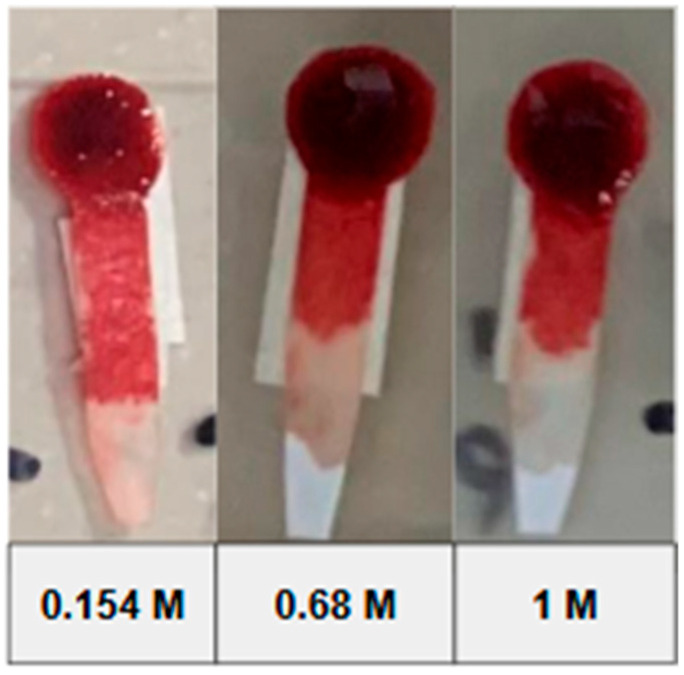
Tests using different concentrations of saline solution.

During preliminary tests, shown in Figure 3, mild hemolysis was specifically checked visually and by color uniformity in the separation zone. No red dye or absorbance background was observed in the plasma zone, confirming that 1 M NaCl did not cause any noticeable hemoglobin leakage. Additional tests were performed in microtubes using NaCl concentrations of 0.5, 1, 2.5, and 5 M, as can be seen in Figure S2 in the Supplementary Material. The 0.5 M and 1 M solutions showed no signs of hemolysis, while at 2.5 M and, especially, at 5 M, clear signs of hemolysis were observed, with the latter showing intense reddish coloration in the plasma.

Glucose solutions with concentrations of 2 mM and 6 mM were tested, representing glucose levels under hypoglycemic and hyperglycemic conditions. The results demonstrated a direct correlation between glucose concentration and the intensity of the color generated in the enzymatic reaction, confirming the device’s sensitivity in measuring glucose levels.

Following the measurement and color reaction, the results were analyzed by processing images of the test paper captured with the smartphone using the specially designed app. For optimal image acquisition, the test paper was placed under good lighting conditions without using the flash, which significantly improved the distinction between the colored areas and enhanced the sensitivity and accuracy of the assay. The analysis was performed using the HSV color model, which was chosen after preliminary comparisons with the RGB model because it offered better discrimination of hue variations during the *TMB* color transition (from blue to green) and was less affected by variations in light intensity. A semi-automatic calibration was performed. This consists of manually selecting the samples of interest within the expected color range and determining appropriate HSV (Hue, Saturation, Value) ranges that allow reliable color to define the color boundaries. Using these parameters, the OpenCV library automatically constructs the complete expected color range. This process enables the system to perform color detection efficiently by generating a binary mask that includes all pixels whose HSV values fall within the specified limits. Consequently, the calibration step ensures that the target color is accurately segmented, even under varying illumination conditions.

It is important to note that no external or internal color reference was used during image capture, so the color measurements depended entirely on the software implementation and the consistency of the imaging conditions, highlighting the role of the app in standardizing image processing.

**Figure 4:**
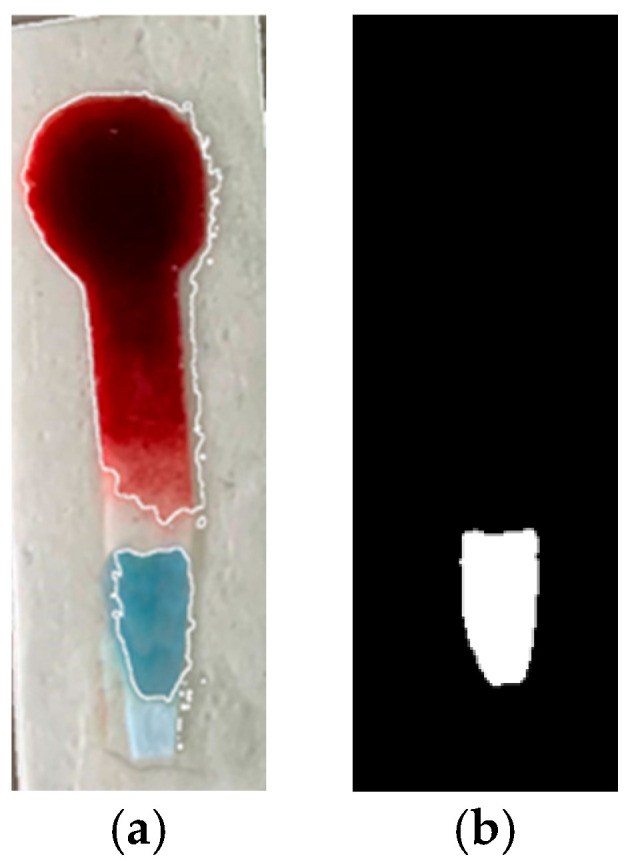
(**a**) Outline of the desired object with the different color areas detected, red: Blood, dark blue: reaction zone; (**b**) Gray mask.

As was mentioned in Step 2 of the Materials and Methods, once the areas are detected, those corresponding to the desired color are shown in white, and the areas that do not match are shown in black, as can be seen in Figure 4.

The measurement protocol consists of collecting an 8 μL blood sample; waiting 10 min; performing the test in a well-lit area, preferably under cold LED lighting or daylight; and taking the photo from a distance of 10 cm without using a flash.

To quantify the flow rate until the reaction is complete, two stages are considered. The first stage runs from the start of the channel to the start of the reaction zone (10 mm channel). The second stage comprises the reaction zone, where the flow rate is measured until the color change is complete. In Whatman No. 1 paper modified with sodium heparin, the capillary flow typically in the first stage is 286.5 ± 15.2 s, corresponding to an average flow rate of 0.035 ± 0.002 mm s^−1^. The total separation was completed in 538.8 ± 109.4 s, corresponding to a total average flow rate of 0.019 ± 0.004 mm s^−1^.

**Figure 5:**
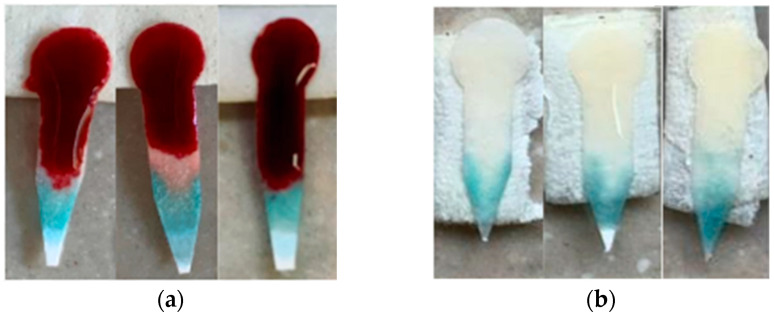
(**a**) Whole-blood samples where the reaction zones are clearly visible in blue; (**b**) plasma samples with the reaction zones in blue.

Calibration is performed through a linear regression between the color intensity recorded by the smartphone and the glucose concentration obtained with the commercial blood glucose test kit. The obtained calibration equation allows the app to adjust an unknown glucose concentration based on color intensity. Glucose determination in the lab is normally determined by using plasma samples. To evaluate if the measurement and the application are consistent when using either whole blood or plasma, a parallel evaluation was conducted. Data collected from several samples (whole blood (a), and plasma (b) from the same sample, Figure 5) in triplicate are organized in a table that includes the glucose concentration measured with the blood glucose test kit, the average of the three color intensities detected for each sample, and the standard deviation of each measurement, as can be seen in Table 1. Each sample was analyzed using the smartphone application. A scatter plot and a calibration equation were obtained by linear regression. Figure 5 shows the resulting chips for each measurement. The non-uniform color distribution observed, even with chitosan modification, can be attributed to several factors. Although the solutions are applied manually on paper, care is always taken to place the drop in the same spot. The porosity of the paper and the viscosity of the different solutions can lead to uneven distribution. Yet, the rapid solvent evaporation can exacerbate these differences by causing uneven reagent spreading and drying. On the other hand, it can be observed that the area of the greatest color forms a V shape following the shape of the paper at the tip. This is not a problem for determination with the smartphone application, as it detects and segments the region of greatest intensity.

The chitosan film (1 mg/mL in 0.25% acetic acid) forms a weakly acidic matrix that stabilizes GOx and HRP, maintaining an effective local pH of ≈6–6.5, within their optimal range. During the tests, only the separated serum was applied to the reaction zone, which does not change pH value. Wang et al. studied the activity of the enzyme immobilized in chitosan with buffer at different pH levels by evaluating the reaction with *TMB*, and concluded that, for glucose detection, color intensity reached the highest value when the pH was 6.0 [25].

**Figure 6:**
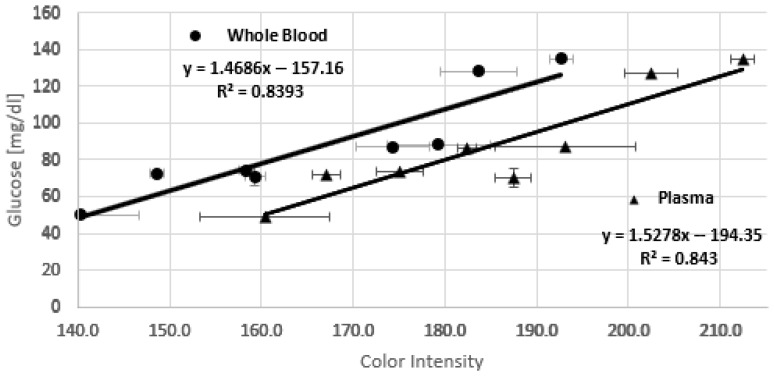
Calibration line of glucose concentration vs. color intensity measured with the whole-blood samples and plasma samples.

The resulting equation allows the application to predict the glucose concentration of a sample simply from the color intensity values, making the system self-contained and efficient for future analysis. Figure 6 shows the calibration curves for whole blood and plasma, respectively. The large error bars observed in some points of Figure 6 likely result from a combination of experimental and material-related factors affecting color uniformity and reproducibility across test strips, as was discussed previously.

The following equations describe the behavior of glucose concentration as a function of color intensity for whole blood (1) and plasma (2) samples.

(3) Glucose [mg/dL]=1.4686×Color Intensity Whole Blood−157.16

(4) Glucose [mg/dL]=1.5278×Color Intensity Plasma−194.35

As can be seen, the lines are parallel, i.e., they have almost the same slope, but there is a constant offset. It is noticeable that the chip overestimates the color intensity, possibly because, in the case of whole blood, the serum is not completely filtered. This issue could be related to the fact that the pore size of Whatman No. 1 paper is larger than the diameter of red blood cells, allowing some of them to pass through and mix with the serum. Additionally, it is important to note that at high salt concentrations, red blood cells could rupture, releasing hemoglobin and generating a faint pinkish color that interferes with the measurement by intensifying the final color.

**Figure 7:**
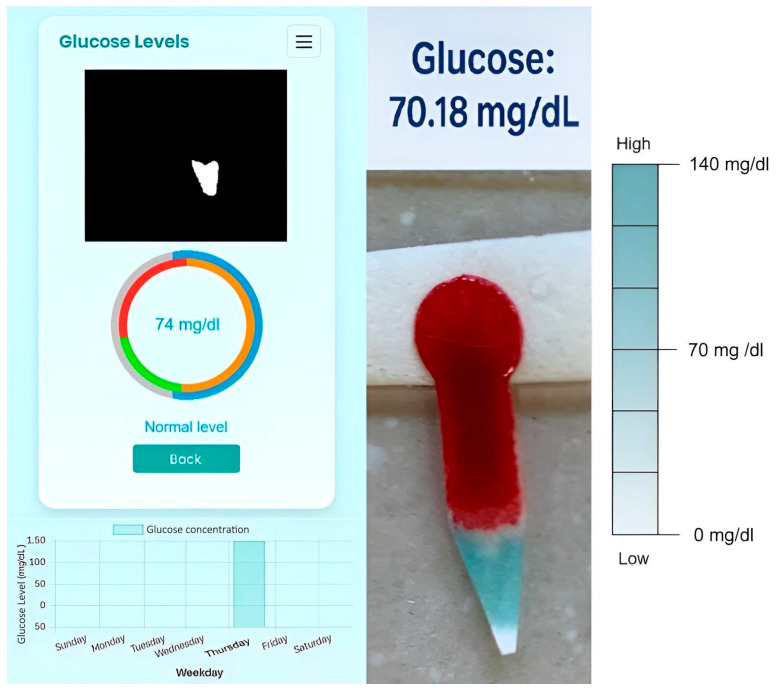
Control and analysis of the application using a blood sample. Colorimetric glucose analysis using the paper strip and digital image processing. The blue region represents the enzymatic reaction proportional to glucose concentration. The example shows a normal blood glucose level (~70–110 mg/dL). The external blue circle in the app fills up to the green zone (normal) and has a light blue. The blue scale on the right is the visual comparison scale for the different glucose concentration ranges in case of not having a Wi-Fi connection or access to a mobile phone.

To evaluate the performance of the system, it was tested with an unknown blood sample. The separated plasma was analyzed using a commercial glucose determination kit, in order to compare the results with those obtained from the developed device. This comparison is shown in Figure 7.

To evaluate the error in the curves, we used the RMSE (root mean square error), a metric widely used to measure the accuracy of predictions. It was calculated for both curves, and its value was approximately 10.83, while the standard deviations of the different subsets of data ranged from 1.5 to 15.4. Since the RMSE is lower than the maximum standard deviation (15.4), it can be inferred that the model presents a reasonable average error compared to the overall variability of the data. However, the RMSE exceeds some of the lower standard deviations, suggesting that the model may have less precise fitting for certain subsets with lower variability. Overall, these results indicate that the model has acceptable performance and adequate predictive capability, although it could benefit from further adjustment to improve accuracy in cases with lower dispersion.

A clear linear trend is observed in both calibration curves, with similar R^2^ and RMSE values, confirming that the measurements obtained with the device using whole-blood samples are comparable to those obtained with plasma.

The detection range of the glucose test strip extends from approximately 50 to 140 mg/dL, as shown in the calibration curves for both whole blood and plasma samples. In terms of stability, the test strips can be stored for up to one week in a freezer (−20 °C), maintaining both chemical and colorimetric stability. When stored at room temperature (20–25 °C), the strips remain stable for at least one day if stored in a cooler, in a dry environment, protected from light and moisture.

## 4. Conclusions

In this work, an innovative method for the separation and analysis of plasma from whole blood was developed and validated, employing a paper-based chip with optimized geometry and surface functionalization. The final design incorporated a 4 mm diameter sample collection zone and an analysis zone connected by a 10 mm long and 2 mm wide channel, enabling reproducible formation of a suitable reaction area for colorimetric glucose detection. Calibration with a commercial kit yielded a correlation of R^2^ = 0.83, indicating reasonable agreement with standard analytical methods. Nevertheless, some deviations were observed between actual and predicted glucose values, which can be attributed to different factors. These can be color non-uniformity, perhaps due to variations in chitosan film thickness, uneven distribution of reactants due to the porosity of the paper, and the viscosity of the different solutions. This problem is overcome to some extent thanks to the image processing used in the smartphone application, as it only detects and segments the region of greatest intensity.

The developed POC device is able to detect samples as small as 8 µL of whole blood. The results demonstrate that the proposed system is a feasible, practical, and cost-effective solution for glucose screening, particularly in low-resource environments. However, they also underscore the need for further optimization to meet the accuracy standards required for clinical applications.

## Figures and Tables

**Table 1:** Glucose concentrations and intensities with whole-blood samples and blood plasma samples.

N°	Glucose [mg/dL] (Commercial Kit)	Average Intensity of Whole Blood	Standard Dev. Whole Blood	Measured Glucose [mg/dL] (Paper Chip)	Average of Color Intensity of Plasma	Standard Dev. Plasma	Measured Glucose [mg/dL] (Paper Chip)
1	50	140.3	12.7	48.9	160.3	14.0	50.6
2	71	159.3	2.1	76.8	158.0	3.8	47.0
3	87	148.7	1.5	61.2	165.0	3.0	57.7
4	88	158.3	1.5	75.4	187.3	5.0	91.9
5	128	174.3	8.1	98.9	167.0	2.1	60.8
6	72.48	179.3	11.2	106.2	175.0	15.4	73.0
7	74.31	183.7	8.4	112.6	182.3	5.9	84.2
8	135	192.7	2.5	125.8	193.0	2.5	100.5

## Data Availability

The original contributions presented in this study are included in the article. Further inquiries can be directed to the corresponding author.

## Supplementary Materials

The following supporting information can be downloaded at: https://www.mdpi.com/article/10.3390/bioengineering12111250/s1, Figure S1: (A) Polycarbonate membrane over Whatman N°1 with whole blood without and with heparin. (B) Glass fiber membrane with functionalized Whatman N°1 paper with whole blood; Figure S2: Visual evaluation of hemolysis at different NaCl concentrations (0.5, 1, 2.5, and 5 M).
