# Supplementary material for: Paper-Based Device for the Colorimetric Determination of Glucose in Whole-Blood Samples Using a Smartphone
Source: Bioengineering (Basel). 2025 Nov 15;12(11):1250. doi: 10.3390/bioengineering12111250 (PMC12650521; doi:10.3390/bioengineering12111250)
Supplement: Supplementary file 1 [file bioengineering-12-01250-s001.zip › bioengineering-3934563-supplementary.pdf]

bioengineering

MDPI

Article

Paper-Based Device for the Colorimetric Determination of Glucose in Whole Blood Samples Using a Smartphone

Lara B. A. Boga [1,†], Katia Gianni[1,†], Mariano N. Aleman[2], Marcos S. Almirón Arroyo[1], Rossana E. Madrid[1,*]

[1] Laboratorio de Medios e Interfases (LAMEIN), DBI, FACET, Universidad Nacional de Tucumán, and Instituto Superior de Investigaciones Biológicas (INSIBIO), CONICET, Av. Independencia 1800, San Miguel de Tucumán 4000, Argentina; labian2013@gmail.com (L.B.A.B.); ka20_04@hotmail.com (K.G.); chato9595@gmail.com (M.A.A.)
[2] Facultad de Bioquímica, Química y Farmacia, Universidad Nacional de Tucumán, Ayacucho 449, San Miguel de Tucumán 4000, Argentina; mariano_edu@hotmail.com (M.N.A.)
* Correspondence: rmadrid@herrera.unt.edu.ar; Tel.: +54-381-436-4120
† These authors contributed equally to this work.

Abstract

In many clinical settings, there is a great need for rapid, simple, and reliable diagnostic tools for the detection and quantification of various biomarkers. These tools enable early medical decisions, which can significantly influence patient recovery. Paper-based analytical devices (PADs) have become promising platforms for rapid and low-cost diagnostic testing in recent years. Among the most important biomarkers is glucose, a key metabolite involved in numerous physiological processes, which allows for the diagnosis and control of diabetes, the prevention of serious long-term complications such as cardiovascular disease, and the monitoring of the effect of medication, diet, and exercise on sugar levels in these patients. A fundamental step in detecting this marker in laboratories is the separation of plasma from whole blood. Several studies have demonstrated the successful integration of plasma separation in μPADs. This work presents the development of a paper-based device for the colorimetric detection of glucose in whole blood samples, allowing plasma separation and using a smartphone to perform a quantitative determination.

Keywords: POC devices; paper-based biosensors

Academic Editor: Firstname Lastname

Received: date
Revised: date
Accepted: date
Published: date

Citation: To be added by editorial staff during production.

Copyright: © 2025 by the authors. Submitted for possible open access publication under the terms and conditions of the Creative Commons Attribution (CC BY) license (https://creativecommons.org/licenses/by/4.0/).

Different membrane materials were evaluated to optimize plasma separation: polycarbonate (3 μm pore size), glass fiber (3 μm pore size, 785 μm thickness), and Whatman No. 1 filter paper (11 μm pore size, 180 μm thickness). The polycarbonate membrane retained excessive sample due to platelet accumulation, which blocked capillary flow. The glass fiber membrane showed higher absorption capacity but required larger sample volumes and exhibited slower flow because of its high liquid retention.

When using whole blood, it contains many formed elements, and this probably clogs the pores, which is why it was very difficult for whole blood to flow through those papers. It is important to note that when using whole blood, separation must be rapid to prevent clotting. Therefore, if the flow is stopped by the accumulation of cells, the sample clots and the device is unable to separate the blood. When using heparinized blood, the effect is the opposite. Everything passes through the pores.

The following figure shows the different papers that have been evaluated.

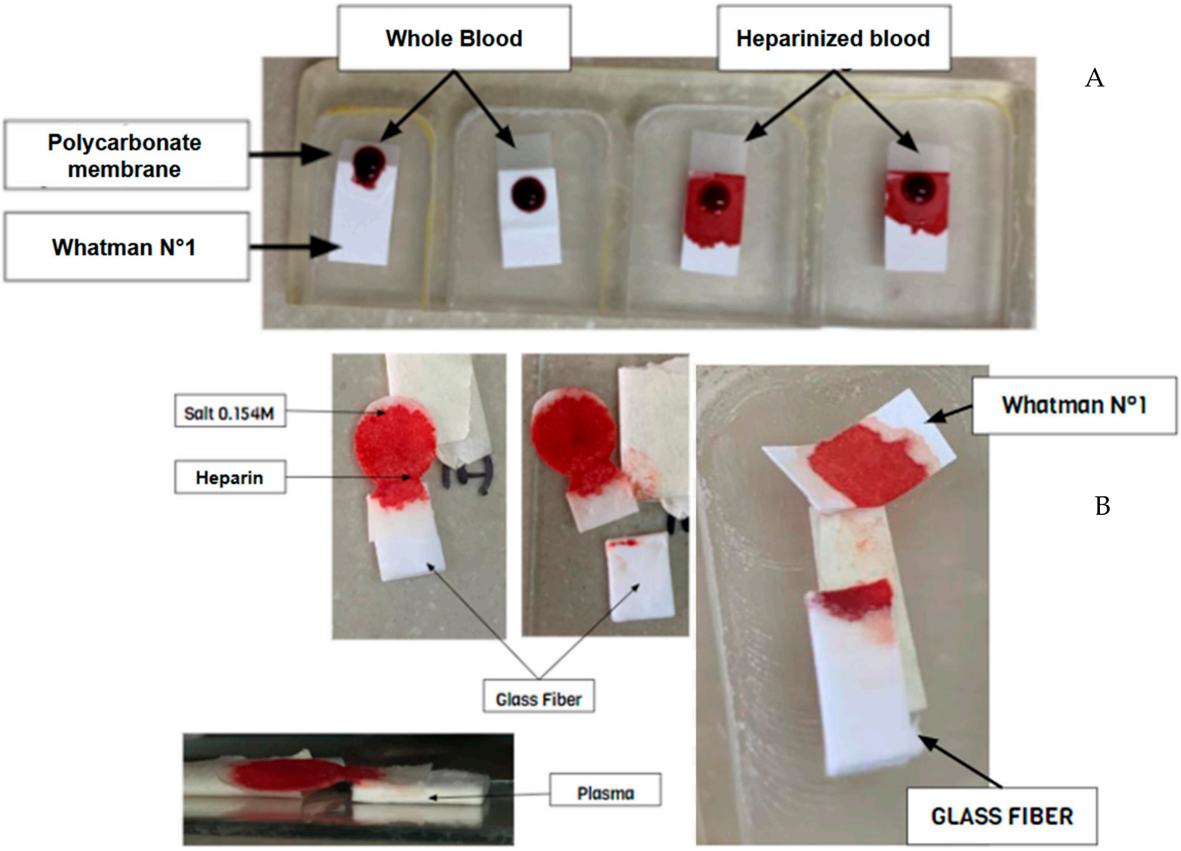

Figure S1. (A) Polycarbonate membrane over Whatman N°1 with whole blood without and with heparin. (B) Glass fiber membrane with functionalized Whatman N°1 paper with whole blood.

For this reason, Whatman No. 1 paper was used despite having a pore size of 11 µm. The use of NaCl allows the erythrocytes, when crenated, to change their globular shape to a structure with spicules. These tend to accumulate and become trapped in the structure of the paper. Nilghaz et al. used, for example, Whatman No. 4 paper, with a pore size of 20-25 µm, and also managed to separate and retain the erythrocytes in the structure of the paper [23]. Whatman No. 1 provided the best balance between capillary velocity and partial cell retention, resulting in a stable flow. This paper proved to be the most efficient compared to the other materials evaluated.

During preliminary tests, shown in Figure 3, mild hemolysis was specifically checked visually and by color uniformity in the separation zone. No red dye or absorbance background was observed in the plasma zone, confirming that 1 M NaCl did not cause any noticeable hemoglobin leakage. Additional tests were performed in microtubes using NaCl concentrations of 0.5, 1, 2.5, and 5 M The 0.5 M and 1 M solutions showed no signs of hemolysis, while at 2.5 M and, especially, at 5 M, clear signs of hemolysis were observed, with the latter showing intense reddish coloration in the plasma.

The following figure shows the different NaCl concentrations that have been evaluated.

 https://doi.org/10.3390/bioengineering12111250

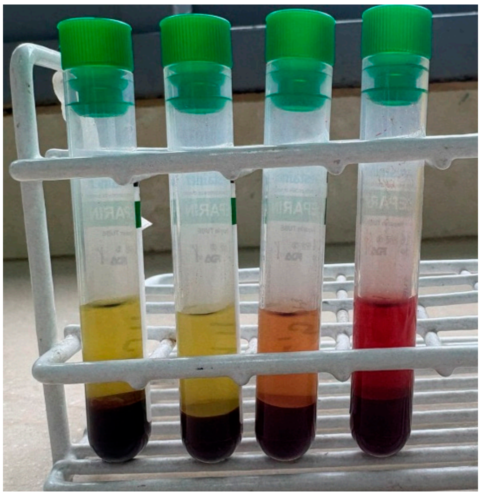

Figure S2. Visual evaluation of hemolysis at different NaCl concentrations (0.5, 1, 2.5, and 5 M).

 https://doi.org/10.3390/bioengineering12111250
